# Propagation Mapping Wave Collision Correlates to the Site of Successful Ablation During Voltage Mapping in Atrioventricular Nodal Reentry Tachycardia

**DOI:** 10.19102/icrm.2017.080905

**Published:** 2017-09-15

**Authors:** Amy Van Aartsen, Ian H. Law, Jennifer R. Maldonado, Nicholas H. Von Bergen

**Affiliations:** ^1^Division of Cardiology, Department of Pediatrics, The University of Wisconsin-Madison, Madison, WI; ^2^Division of Cardiology, Department of Pediatrics, The University of Iowa, Iowa City, IA

**Keywords:** Ablation, atrioventricular nodal reentry, three-dimensional mapping, propagation mapping, voltage mapping

## Abstract

Voltage mapping has been used previously for slow-pathway localization for atrioventricular nodal reentrant tachycardia (AVNRT) ablation. However, propagation mapping may be a technique to further improve the localization of the slow pathway. This retrospective study aimed to evaluate the relationship of the propagation map to both the voltage mapping and successful site of ablation in patients who underwent ablation for AVNRT. All patients ≤20 years of age who underwent voltage mapping for AVNRT were included in this study. Patients were excluded if they had congenital heart disease or inadequate voltage point density within the triangle of Koch (TK). During the study, a propagation map was evaluated from the prior voltage map, marking a “wave collision” at the site of atrial wave convergence. Patient and procedural information, the location of the wave collision, the site of successful ablation, and the appearance of the voltage map were evaluated. Ultimately, 39 patients aged from four years of age to 20 years of age were evaluated. Success was achieved in 100% of patients, with a recurrence rate of 2.8% and no long-term complications observed. The average procedure time was 127 min. Follow-up length averaged seven months post operation. Low-voltage areas, and a wave collision, were present in all patients. This wave collision was typically located within the TK. The median number of ablations required for successful outcome was two. The successful ablation lesion was typically located over a low-voltage area within 4 mm of the wave collision within the TK. In conclusion, we found in this retrospective evaluation that propagation mapping resulted in a wave collision within the TK, and that the successful ablation site in the majority of patients was near a low-voltage area within 4 mm, typically superiorly, to the wave collision within the TK.

## Introduction

Atrioventricular nodal reentry tachycardia (AVNRT) is the most common type of reentrant supraventricular tachycardia (SVT), and is one of the most prevalent arrhythmias in the pediatric and young adult population.^1,2^ In recent years, voltage mapping has been utilized as a technique to identify the slow pathway to guide the ablation of AVNRT.^2–4^ However, although the voltage mapping technique facilitates slow-pathway localization in the majority of patients, the rationale to improve upon this technique exists, as not all patients present a characteristic low-voltage target, and subjectivity is required when evaluating an individual’s voltage map.^4^

Propagation mapping, when used in conjunction with voltage mapping, may further improve the localization of the slow pathway. This retrospective study aimed to evaluate the relationship of the propagation map to both the voltage map, and the successful site of ablation, in patients who underwent an ablation procedure for AVNRT.

## Methods

All patients aged 20 years and younger with AVNRT who underwent voltage mapping for evaluation and guided ablation between January 2010 and May 2016 at two institutions were enrolled in this study. Patients were excluded if they had congenital heart disease. The institutional review boards (IRBs) at both hospitals approved this study’s protocol, and the study was performed in accordance with the relevant portions of the Declaration of Helsinki. Data collected included patient demographics and procedural characteristics, including the appearance of intracardiac voltage mapping, the duration of the procedure, any complications, the number of ablations needed to achieve success, the total number of ablations, follow-up evaluation information, and recurrence rate. For each patient, a previously obtained three-dimensional (3D) electroanatomical map was evaluated for the number of voltage points, and reevaluated with the addition of propagation mapping (which was not consistently done at the time of the procedure). If there was inadequate voltage point density for interpretation of the voltage or propagation maps, then such was excluded from further analysis. No patient consent was obtained for inclusion in this study, due to the need for it being waived by the IRBs.

### Procedural technique

The procedural technique for voltage mapping has previously been described and is briefly reviewed here.^3^ In all procedures, a 3D construction of the endocardial geometry of the right atrium and coronary sinus was created using the EnSite™ NavX™ or EnSite™ Velocity™ cardiac mapping system (Abbott, Chicago, IL, USA). Once AVNRT was identified, a voltage map was acquired. During sinus rhythm, the peak-to-peak atrial amplitude voltage points were collected, using the 3D atrial endocardial geometry and contact mapping of the atrial signals. This was typically acquired using a steerable diagnostic catheter with focused data collection, within the triangle of Koch (TK). Ectopic beats were excluded from analysis. During the procedure, the high-voltage and low-voltage sliders were typically initially set at 2.0 mV and 0.2 mV, respectively, to assist with either low- or high-voltage differentiation of the atrial tissue. The low-voltage slider bar could then be adjusted, typically between 0.1 mV and 0.5 mV to assist with the identification of low-voltage areas if desired. The low-voltage areas of interest were previously described by Bailin et al. as a low-voltage bridge between higher-voltage areas, or as a broad low-voltage area connecting the low TK to the atrioventricular (AV) nodal area.^3^

In slight contrast to Bailin et al., our operators defined the low-voltage area of interest somewhat more broadly, as an area of low voltage within the TK that was not specifically dependent on a bridge appearance. Areas were typically considered low voltage if they were less than 0.5 mV. Once identified, these low-voltage areas could then be targeted for ablation. Cryotherapy was initially used in all patients because of physician preference and, when possible, the ablation was completed during AVNRT presentation to assess the time-to-effect, as shown by the slowing and termination of the AVNRT. If there was no effect within 20 to 30 s of cryothermal application, the initiation of therapy was stopped. At the successful site, a four-to-five-minute application of therapy (-70° C) was placed. Subsequently, additional insurance options were placed in the area surrounding the successful site. If an echo beat remained present after termination of the AVNRT pathway, additional test applications were placed using anatomical guidance (as opposed to targeting a low-voltage site) within the TK in an attempt to eliminate all residual slow-pathway conduction. Nevertheless, as long as no more-than-single echo beats existed, then the procedure was considered to be a success. In some of the later cases, the propagation map was also evaluated at the time of the procedure, though the selection of the ablation site was still performed using the voltage map as described above.

### Post-procedure processing

**Figure 1:**
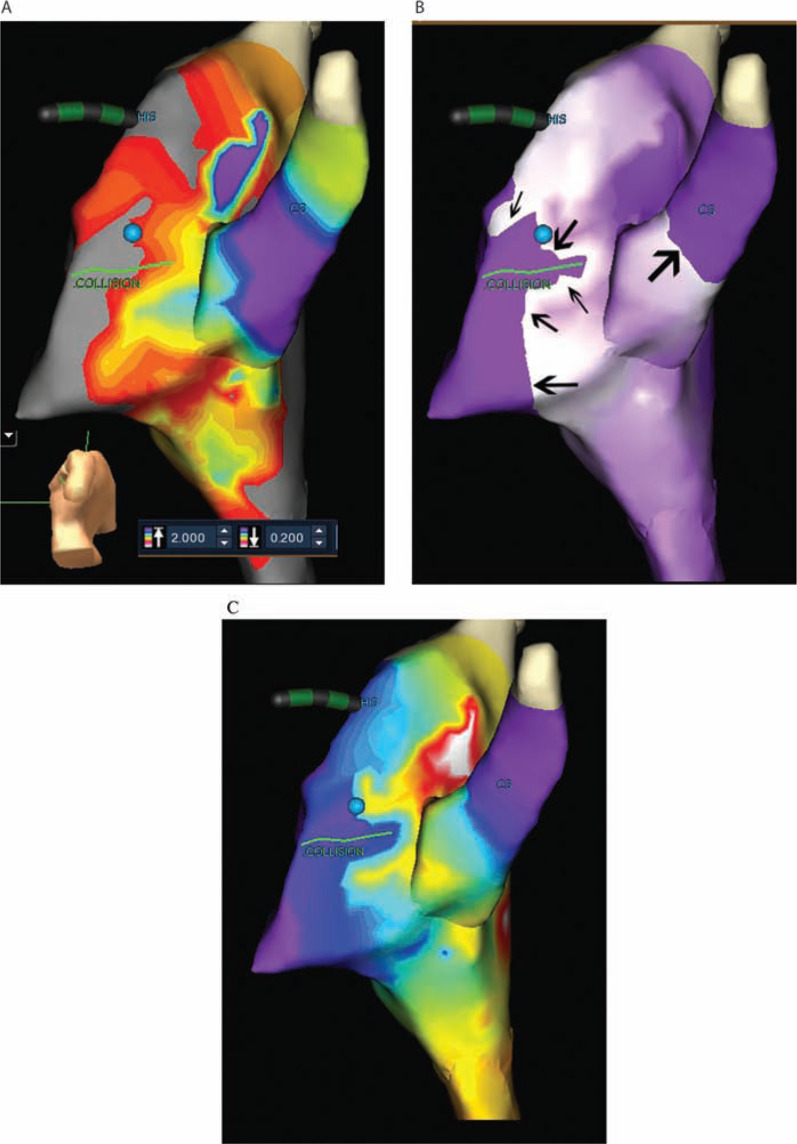
**A:** An example of a left lateral, slightly posterior/inferior, view of the atrial septum displaying a voltage map. Note that the His-bundle markers (“HIS” in blue), as well as the CS, are designated. The blue sphere represents the successful site of cryotherapy. The wave collision is marked as the green line just above the “.COLLISION” label. The light gray area on the atrial surface represents a voltage < 0.2 mV, with the color gradient changing to purple with voltages > 2.0 mV. **B:** This figure is the same geometry as **A** but with a still frame of the propagation map. The green line represents the marked wave collision, just above the label of “.COLLISION.” The white represents the wave propagation, with the wave front on the purple atrial geometry, proceeding in the direction of the arrows. The wave fronts collide at the marked wave collision. The His-bundle (“HIS” in blue) and the CS are labeled. **C:** This figure uses the same geometry as seen in **A,** but with the propagation map displayed as an activation map. Each change in color represents a consistent segment of time of atrial activation, from earlier (in white) to later (in purple). The more pronounced the changes in activation map color, the slower the conduction. Note the slower conduction through the superior TK, with a more rapid conduction posterior to the CS and superior to the site of the wave collision.

In all patients, the voltage points were evaluated and excluded if they were felt to be inaccurate (such as during a premature atrial complex or paced beat). The voltage map was evaluated to confirm that there was an adequate point density within the TK (at least 30 points in the TK). Subsequently, the remaining voltage points were utilized to evaluate the propagation of the atrial signal during sinus rhythm and the voltage map. As this was a retrospective evaluation, patients were excluded from the evaluation if it was determined that there were inadequate voltage points to allow for the interpretation of either the voltage map or the propagation map. In an attempt to standardize the technique, the low-voltage slider bar for the voltage map was set at 0.2 mV, and the high-voltage slider bar was set at 2.0 mV **(Figure 1A)**. The propagation map was then evaluated for the site of the “wave collision.” The wave collision site was considered to be the location of intersection of two or more wave fronts, typically within or around the TK. The sites of the wave collision were marked on the 3D map **(Figure 1B)** and confirmed by two of the authors of the current study. In some of the more recent procedures, the propagation map had been evaluated at the time of the procedure; therefore, the previously selected wave collision site was confirmed with retrospective evaluation. During post-procedure processing, the following parameters were evaluated: the voltage map, the propagation map, the site of successful ablation, the number of surface data points, the presence and site of low-voltage areas, and the presence and site of a wave collision. The relative locations of these parameters were used for analysis. The distance between the successful site and the wave collision was quantitatively measured and defined as the distance from the center of the successful ablation to the nearest point on the wave collision as mapped by the EnSite™ NavX™ system (Abbott, Chicago, IL, USA).

## Results

In total, 72 patients who underwent AVNRT ablation were screened for inclusion in this study; however, of these, 33 patients were excluded because of the presence of congenital heart disease or the lack of an adequate propagation or voltage map to allow for analysis. For the 39 patients finally evaluated, the median age was 15 years (4 to 20 years), and 23 of these patients (59%) were female **(Table 1)**. The average patient weight at the time of the procedure was 64.2 kg (range 19.1 to 119.2 kg).

The mean procedure time was 127 ± 52 min. The median fluoroscopy used for the procedures was 0 s, though 102 s of fluoroscopy was used in the case of one patient during a transseptal procedure for a presumed left-sided AVNRT. Other than this, however, no radiation was used. Procedural success, as defined by no inducible AVNRT and no more than a single AV node echo beat at the conclusion of the procedure, was achieved in all patients. There were no procedural complications observed, although two patients developed a small hematoma from the access site after the procedure, both of which resolved without further complication. There was one recurrence (3%) within the average follow-up period of 7 ± 9 months as measured from the time of the procedure to the most recent appointment. Patients were typically discharged from care after two visits if they had no recurrence of symptoms.

Inducible typical AVNRT was present in 37 of the patients, and inducible atypical (fast-slow) AVNRT was present in three of the patients (one patient presented with both typical and atypical AVNRT). All 39 patients underwent general anesthesia and isoproterenol infusion during the procedure. However, if the arrhythmia was inducible off isoproterenol, then the isoproterenol was typically used only during post-ablation testing. Cryothermal ablation was used based on physician preference as the initial ablation energy source for all of the study patients. In one patient, radiofrequency (RF) energy was used as a crossover after the lack of success with cryotherapy. Interestingly, this patient had the eventual successful RF ablation site located near the first cryoablation site after also being evaluated for a left-sided slow pathway. The successful ablation site was within the first three ablations in 27 (77%) of the 39 patients. The median number of ablations to achieve success was two (range 1–71).

The location of the successful site was over a low-voltage area for all of the patients. The total number of cryothermal ablations was a median of 10 applications (range 4 to 71 applications). For the patient who also received RF application after cryotherapy was unsuccessful, a total of 101 ablations of RF and cryotherapy were placed. The additional applications that were placed following the successful termination of AVNRT were either insurance applications, or those placed in an attempt to ablate an echo beat if it remained present.

During the ablation procedure, nine patients underwent evaluation of the propagation map to determine if there was a wave collision **(Figure 1C**; displayed as an activation map). Each propagation map displayed a wave collision in the TK. However, as we had not yet evaluated any relationship between a wave collision and the successful site of ablation, the propagation wave collision was not used for ablation site selection.

### Propagation mapping evaluation

**Figure 2:**
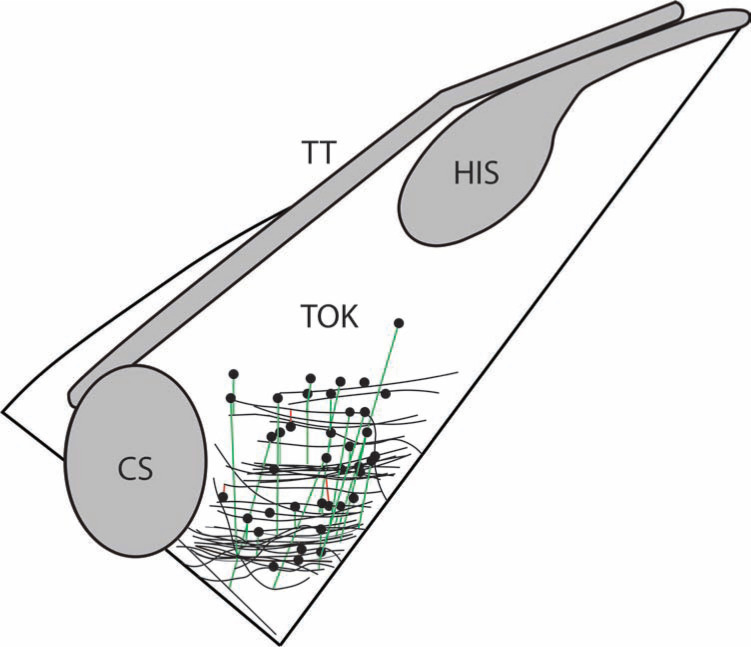
Propagation wave collision and successful ablation site. An illustration of the TK is outlined; His bundle (His), coronary sinus (CS) os, and tendon of Todaro (TT). The wave collision for each patient is represented as a black line. The individual’s wave collision is connected to the site of successful ablation, with green lines connecting the site of the center of a successful lesion, which is closer to the AV node than the wave collision; and red lines connecting successful lesions further from the AV node. In 87% of the patients, the center of the successful ablation site was located on or above the wave collision. Though the successful lesion sites are represented by a small circle at the center of the successful site marked on the 3D map, it should be noted that the typical lesion was 6 mm in diameter, substantially larger than the representative circle seen in this figure.

**Figure 3:**
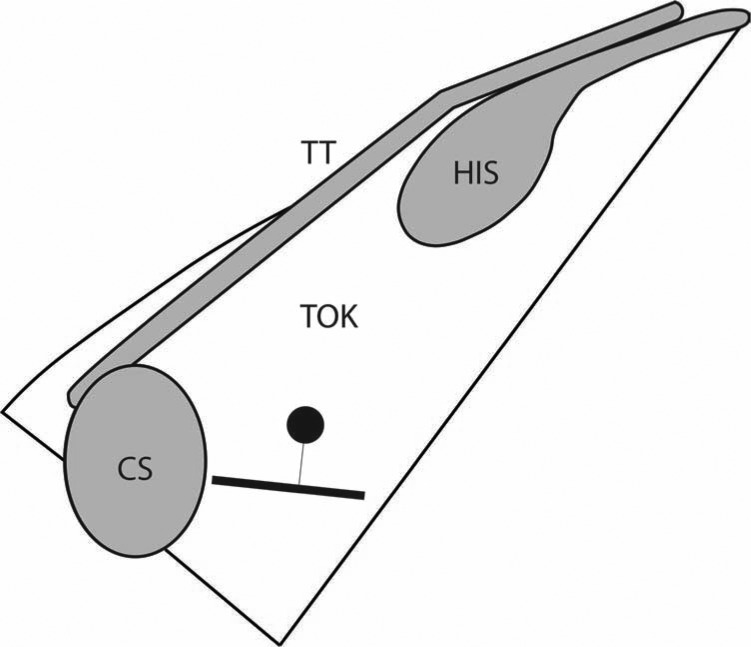
The estimated average wave collision and ablation site. This figure shows a representation of the TK and the estimated average site of wave collision and location of successful lesion creation.

A low-voltage area in the TK, which may represent a slow-pathway target for ablation, was present in all patients. When evaluating the propagation map, a wave collision was present on the propagation map for all of the patients **(Figure 1B)** during an atrial-driven rhythm (typically sinus). The most typical appearance found two wave fronts colliding near the mid-TK. Most commonly, there was a slow progressive wave front through the high TK, with a concurrent higher velocity wave front passing posterior to the tendon of Todaro and progressing around the posterior coronary sinus (CS) to begin an inferior-to-superior TK progression. The propagation of the atrial signal is displayed in **Figure 1C,** illustrating the conduction of the atrial signal both superior-to-inferior and inferior-to-superior within the TK, with a wave collusion occurring in the mid-TK. For each patient, the location of the wave collision was marked on the 3D map and evaluated in comparison with the successful site of ablation and the low-voltage areas **(Figures 2 and 3)**.

The wave collision was typically within the TK, around the level of the mid-CS. In 87% of the patients, the center of the successful ablation site was located on or higher in the TK than the wave collision. In 69% of patients, the center of the successful ablation site was within 4.0 mm of the wave collision (range 0 to 15.5 mm). It should be noted that the cryolesions were typically performed with a 6.0-mm-tip catheter, which may result in the overlap of the wave collision and the ablation in many cases.

There did not seem to by any association between the location of the wave collision and typical or atypical AVNRT. In the patient with crossover to RF, the successful site was located directly on the wave collision in the lower region of the TK, though cryotherapy did not terminate the arrhythmia during the initial test applications. This was also the only patient with received fluoroscopy, as a transseptal was performed to evaluate for a left-sided AVNRT pathway.

## Discussion

The use of voltage gradient mapping to visualize the slow pathway has been shown to allow for the success of electrically guided ablation of AVNRT.^3,4^ In place of a purely anatomically guided approach, this approach has theoretical benefits, including the potential to decrease partial ablations, reduce procedural times, and identify possible ablation sites that are further from the AV node.^4^ However, one relative disadvantage of voltage mapping is the subjectivity of the low-voltage areas. In particular, many patients will have multiple low-voltage areas within the TK, resulting in multiple sites, which could be considered suitable slow-pathway ablation. In an attempt to standardize the analysis, we set the low-voltage slider bar at 0.2 mV and the high-voltage slider bar to 2.0 mV. This standardization of the voltage settings did not appear to affect our ability to evaluate for low-voltage areas. By decreasing the subjectivity, we hope to make this technique useful to a broader scope of practitioners including those with limited experience with voltage mapping.

Even with the use of the voltage-guided approach, there are still challenges to AVNRT ablation that are concerning, including difficulties with mapping, procedural risk, and post-procedure recurrences. This suggests the need for continual improvement of AVNRT ablation techniques.

Prior studies evaluating voltage mapping did not find and observable voltage “bridge” in all patients.^2–4^ In contrast to prior reports, which relied on the concept of the type I or type II voltage bridges as described by Bailin et al., in this study we considered a low-voltage area within the TK as a potential slow pathway, and we found low-voltage areas in all patients.^3^ It should be noted that in an attempt to provide clean and accurate data, we were fairly rigorous about excluding patients with fewer than 30 voltage points in the TK, or those who have inadequate point density with which to evaluate propagation mapping. This combination likely increased our likelihood of finding a low-voltage area and the occurrence of subsequent wave collision.

To our knowledge, our study is the first to evaluate the relationship of a propagation wave collision to the voltage map and the site of successful ablation. As this is a retrospective study, we acknowledge the potential limitations. In particular, as the majority of procedures did not incorporate propagation mapping at the time of ablation, it is unknown if propagation mapping will improve success, decrease recurrence, or guide the ablation to sites outside of the typical ablation sites. Nevertheless, even with these limitations, these are the first data to suggest a correlation with the wave collision, low-voltage areas and the location of the successful ablation site. In particular, the successful ablation site is often on or slightly superior (towards the AV node) in location compared with the propagation wave collision point within the TK. Given the observed correlation among the wave collision, the successful ablation site, and a low-voltage area, these findings suggest that propagation mapping may improve slow-pathway localization. As mentioned above, the successful site was generally on or superior to (87%) and within 4.0 mm of (69%) the wave collision. Future studies, some of which are currently underway, will include a prospective evaluation of this technique. As with other studies, data from this study found that the atrial voltages were low at the successful ablation site.^2–4^

Consistent with previous studies on AVNRT, as multiple wave fronts approach from different directions, it would be expected to cause a wide, fractionated, low-voltage electrogram.^5,6^ Other studies have shown alterations in timing, electrogram amplitude, and His-bundle appearance in the perinodal area, depending on the fast- or slow-pathway conduction. In particular, Zhang showed that the transition from fast-pathway conduction to slow-pathway conduction was associated with the formation of different wave fronts with the TK.^7^ In this study, the wave fronts changed with higher heart rates, demonstrating more sluggish conduction from the superior TK area. This may have implications in the interpretation of a wave collision as a marker for slow-pathway ablation, as varying heart rates may play some part in altering the site of atrial wave collision. Unfortunately, alterations in the wave collision site with different heart rates were not evaluated in this study. In recent practice, we have often found that the areas beneath the wave collision were associated with brisker conduction, had higher voltages, and were less likely to have a fractionated atrial signal; these are areas typically considered unlikely for slow-pathway conduction.

### Study limitations

This study is limited by its retrospective nature as highlighted above. In particular, although this study suggests correlations between the propagation mapping wave collisions, low-voltage areas and the site of successful ablation therapy, a prospective study will be needed to draw conclusions regarding the effectiveness for propagation mapping in this population.

## Conclusions

In this retrospective evaluation, we found that propagation mapping revealed a wave collision within the TK. The successful ablation site in the majority of patients was within the TK on a low-voltage area on or within 4.0 mm superior to the wave collision.

## Figures and Tables

**Table 1:** Procedural Information

Average procedure time	127 ± 52 min.
Median fluoroscopy time	0 s (one patient with 102 s).
Inducible AVNRT	100%.
Complications	No procedural complications.
Average length of follow-up	7 ± 9 months.
Recurrence	3% (one patient).
Median ablations performed until success	2 (range 1–71).
Median total number of ablations performed	10 (range 4–103; included RF therapy).
Typical/atypical AVNRT	36:2; one both.
